# The burden of acute kidney injury - a cross-sectional research in an university intensive care unit

**DOI:** 10.1186/2197-425X-3-S1-A733

**Published:** 2015-10-01

**Authors:** T Bergler, E Bossauer, K Thelen, BM Graf, B Banas, HJ Schlitt, W Gnann, T Bein, I Gocze

**Affiliations:** University Medical Center Regensburg, Dep. of Nephrology, Regensburg, Germany; University Medical Center Regensburg, Dep. of Corporate Development, Regensburg, Germany; University Medical Center Regensburg, Dep. of Surgery, Regensburg, Germany; University Medical Center Regensburg, Dep. of Anesthesia, Regensburg, Germany

## Introduction

The prognosis of intensive care patients with acute kidney injury (AKI), which is associated with increased mortality rates (up to 30 %), is still poor. In the long run, patients with acute kidney injury on ICU have a high risk of maintaining advanced chronic kidney disease (CKD) or even end-stage renal disease (ESRD).

## Objectives

The aim of this study was to investigate the prevalence of AKI in adult critically ill patients in the University Hospital, Regensburg, Germany. Furthermore, we searched for the most common ICU conditions associated with AKI, evaluated the length of stay in the intensive care unit (ICU) and in the hospital and calculated the additional in-hospital costs associated with AKI in Diagnosis Related Groups (DRG) based reimbursement system in Germany.

## Methods

In this retrospective study all finished admissions in 5 adults ICUs during 2011-2013 were evaluated. AKI and the 3 ICU-diagnosis commonly associated with AKI were identified using ICD-10 classification. Using regression analyses, the length of stay in hospital and ICU for patients with and without AKI was examined. AKI-associated in-hospital costs were calculated for 2013 according to DRG-related calculation for single diagnosis/ procedure and adjusted to nationwide base case value for reimbursement.

## Results

In total 891 ICU-patients AKI was classified according to ICD-10 code. Acute respiratory distress syndrome (ARDS), myocardial infarction (MI) and sepsis were the three most common ICU-conditions associated with AKI. 1103 patients were admitted with one of these three diagnoses during 2011-2013 and 249 (22.6%) of these developed an AKI. Patients with AKI had a significantly longer ICU and hospital stay compared to patients without AKI (18.6 vs 5.1 days and 23.8 vs. 10.4 days, p < 0.001). In-hospital costs for septic patients with AKI were higher than for septic patients without AKI (25 216 € vs. 13 486 €). Patients with ARDS or MI without AKI had an in-hospital costs of 12 387 € compared to 41 949 € for patients with ARDS or MI and AKI. Presence of AKI in critically ill patients with ARDS, MI or sepsis was associated with additional costs of 2 019 120 € in the University Hospital, Regensburg in 2013.

## Conclusions

Occurrence of AKI in critically ill patients is associated with significant longer ICU- and hospital stay. Moreover, it causes considerable higher in-hospital costs in DRG-based reimbursement. Early recognition of patients at risk, coordinated research for novel interventions and establishment of national AKI-network for implantation of evidence-based therapies may decrease the incidence of AKI, improve outcome and save costs for national health care system.

